# NEARER SCAN (LENO BESIK) evaluation of a task-sharing echocardiographic active case finding programme for rheumatic heart disease in Australia and Timor-Leste: protocol for a hybrid type II effectiveness-implementation study

**DOI:** 10.1136/bmjopen-2023-083467

**Published:** 2024-10-18

**Authors:** Benjamin Jones, James Marangou, Jennifer Yan, Anna Ralph, Alice Mitchell, Alex Kaethner, Bo Remenyi, Vicki Wade, Judith M Katzenellenbogen, Anferida Fernandes Monteiro, Jeffrey W Cannon, Natasha J Howard, Marisa Gilles, Emma Haynes, Herculano Seixas, Joaquina Maurays, Jade Neave, Chantelle Pears, Daniel Engelman, Karla Canuto, Andrew Steer, Holger Unger, Meghan Bailey, Maria Tanesi, Salvador Amaral, Helder Neto, Maida Stewart, Paul Burgess, Alex Brown, Bart J Currie, Graham Hillis, Peter Morris, David Simon, Gavin Wheaton, Jacqui Williamson, Jessica de Dassel, Simon Slota-Kan, Jonathan Carapetis, Mike English, Shobhana Nagraj, Joshua R Francis

**Affiliations:** 1 Health Systems Collaborative, Nuffield Department of Medicine, University of Oxford, Oxford, UK; 2 Menzies School of Health Research, Charles Darwin University, Darwin, Northern Territory, Australia; 3 Global and Tropical Health Division, Menzies School of Health Research, Darwin, Northern Territory, Australia; 4 Department of Paediatrics, Royal Darwin Hospital, Darwin, Northern Territory, Australia; 5 Heart Foundation, Sydney, New South Wales, Australia; 6 School of Population and Global Health, University of Western Australia, Crawley, Western Australia, Australia; 7 Wesfarmers Centre for Vaccines and Infectious Diseases, Telethon Kids Institute, Nedlands, Western Australia, Australia; 8 South Australian Health and Medical Research Institute, Adelaide, South Australia, Australia; 9 Western Australia Country Health Service, Perth, Western Australia, Australia; 10 University of Western Australia, Perth, Western Australia, Australia; 11 Hospital Nacional Guido Valadares, Dili, Timor-Leste; 12 Maluk Timor, Dili, Timor-Leste; 13 Murdoch Children's Research Institute, Parkville, Victoria, Australia; 14 Flinders University, Adelaide, South Australia, Australia; 15 Department of Medicine, Royal Melbourne Hospital, University of Melbourne, Parkville, Victoria, Australia; 16 Timor-Leste Ministerio da Saude, Dili, Timor-Leste; 17 Miwatj Health Service, Darwin, Northern Territory, Australia; 18 Northern Territory Government of Australia, Darwin, Northern Territory, Australia; 19 Indigenous Genomics, Australian National University, Canberra, Australian Capital Territory, Australia; 20 Telethon Kids Institute, Perth, Western Australia, Australia; 21 Department of Infectious Diseases, Royal Darwin Hospital, Casuarina, Northern Territory, Australia; 22 School of Medicine and Pharmacology, University of Western Australia, Perth, Western Australia, Australia; 23 Katherine Hospital, Katherine, Northern Territory, Australia; 24 Department of Cardiology, Women’s and Children’s Hospital, Adelaide, South Australia, Australia; 25 Department of Public Health and Primary Care, University of Cambridge, Cambridge, UK

**Keywords:** health workforce, implementation science, public health

## Abstract

**Introduction:**

Rheumatic heart disease (RHD) is underdiagnosed globally resulting in missed treatment opportunities and adverse clinical outcomes. We describe the protocol for a study which aims to co-design, implement and conduct an evaluation of a task-sharing approach to echocardiographic active case finding for early detection and management of RHD in high-risk settings in Australia and Timor-Leste.

**Methods and analysis:**

Echocardiograms will be obtained by trained local staff using hand-held echocardiographic devices employing the ‘Single Parasternal Long Axis view with a Sweep of the Heart’ (SPLASH) technique and interpreted by experts remote from the site of acquisition. Approximately 1500 children and pregnant women will be screened across high-risk communities in Australia and Timor-Leste over an 18-month period. The study will use a type II effectiveness-implementation hybrid design. A tailored package of implementation strategies will be co-designed with communities and health services and mapped onto a Theory of Change framework. The clinical effectiveness will be assessed as the change in the proportion of the target population that are prescribed secondary prophylaxis for RHD by the end of the study compared with baseline. The implementation will be assessed as the adoption, penetration, sustainability, fidelity and cost of the programme with a mixed-methods theory-based and economic evaluation. Data will include numbers of normal, abnormal and uninterpretable SPLASH echocardiograms obtained, numbers of participants progressing through the cascade of care, interviews with staff and programme costs.

**Ethics and dissemination:**

Ethical approval has been obtained from the Human Research Ethics Committee of the NT Department of Health and Menzies School of Health Research, Darwin (HREC-2022-4479), the Western Australian Aboriginal Health Ethics Committee (HREC-1237) and the Instituto Nasional Saude Publika Timor-Leste Ethics and Technical Committee (03-UEPD/INSP-TL/V/2023). Informed consent is required to be enrolled. Study findings will be disseminated in the communities involved and submitted for publication.

**Trial registration number:**

NCT06002243.

**STRENGTHS AND LIMITATIONS OF THIS STUDY**

- A type II effectiveness-implementation hybrid design allows simultaneous evaluation of the intervention’s implementation strategy and clinical effectiveness.
- The co-design approach ensures that the intervention is tailored to local contexts, enhancing likelihood of successful implementation.
- The use of theory ensures the implementation of the programme uses existing evidence.
- The study involves two countries (Australia and Timor-Leste), enhancing the generalisability of findings.
- The primary outcome may be influenced by external factors beyond the intervention.

## Introduction

 Rheumatic heart disease (RHD) is a preventable, acquired cardiac condition. It occurs as a complication of repeated childhood infections with *Streptococcus pyogenes* which trigger acute rheumatic fever (ARF). There are estimated to be over 40 million people living with RHD globally and more than 300 000 RHD-related deaths per year.^1^ The highest rates of RHD are reported in sub-Saharan Africa, South Asia and the Oceania region, including Timor-Leste^2^ and the First Nations populations in Australia and Māori and Pasifika communities in Aotearoa New Zealand, affecting up to 5% of school-age children in some communities.^3^ RHD is an important indirect cause of maternal morbidity and mortality within at-risk populations,^4^ including Aboriginal and Torres Strait Islander women.^5^ RHD contributes to premature mortality and severe long-term cardiovascular morbidity in these settings,^23 6^,^8^ where clinical presentations are frequently late in the disease process, and opportunities for early treatment are often missed.^9^

Active case finding or screening for RHD meets Australian criteria for population screening,^10^ and is justified by evidence that clinical outcomes can be improved if RHD is detected early and secondary prevention with antibiotic prophylaxis is initiated.^11 12^ Echocardiography is more accurate than auscultation.^13^,^16^ Programmes using echocardiography consistently identify cases of asymptomatic RHD that would have otherwise been missed.^1314 17^,^20^

The recently published 2023 World Heart Federation (WHF) guidelines for the echocardiographic diagnosis of RHD provide a gold standard for RHD diagnosis, in addition to screening criteria for non-expert conducted hand-held echocardiography screening.^21^ Building on the original 2012 WHF guidelines for echocardiographic diagnosis of RHD, the 2023 guidelines incorporate the emergence of innovative solutions to overcome the challenges of limited trained experts and costly equipment. These solutions include: (1) task-sharing of echocardiographic detection between experts and non-experts using more affordable, hand-held echocardiographic devices; and (2) abbreviated screening protocols with adjusted criteria.^22^,^24^

Evidence supporting a task-sharing approach in echocardiographic detection of RHD is derived from diagnostic accuracy studies conducted in endemic regions.^25^,^33^ Task-sharing refers to healthcare tasks being completed collaboratively by healthcare providers with different training.^34^ By contrast, task *shifting* differs in that the task is completely transferred to a different cadre of the workforce. Early studies of task *shifting* for echocardiographic detection involved nurses who were trained to perform echocardiography and interpret images.^26 30^ Our work to date in Australia and Timor-Leste has identified superior diagnostic accuracy with task-sharing, whereby an expert can review images obtained by a non-expert who has been trained to use a hand-held echocardiographic device to obtain images based on an abbreviated Single Parasternal Long Axis view with a Sweep of the Heart (SPLASH) protocol.^28 35^ The SPLASH protocol aligns with the 2023 WHF screening criteria for the echocardiographic diagnosis of RHD.^21^ We have been able to demonstrate acceptable diagnostic accuracy (sensitivity 76.7% (95% CI 68.5% to 83.7%), specificity 94.9% (95% CI 94.0% to 95.6%)) using this approach.^35^

### Aims

The overall aim of the ‘Non-Expert Acquisition and Remote Expert Review of Screening echocardiography images from Child health and AnteNatal clinics’ (NEARER SCAN; ‘LENO BESIK’ in Tetum) study is to co-design, implement and evaluate a task-sharing approach to echocardiographic active case finding for early detection and management of RHD in high-risk settings in Australia and Timor-Leste.

Specific objectives include:

1. To co-design locally tailored implementation strategies with the clinic and community in each site and to capture this initial logic in a Theory of Change.
2. Based on these co-designed strategies, to implement SPLASH echocardiography training and a task sharing approach to echocardiographic detection for early detection and management of RHD in communities.
3. To conduct a mixed-methods theory-based evaluation to assess the programme adoption, penetration, sustainability and fidelity, and to explain what implementation strategies assist in integrating this task-sharing approach into existing health services, under what circumstances and why.
4. To determine the clinical effectiveness of the intervention in terms of impact on detection and diagnosis of RHD, and linkage to guideline-based clinical care.
5. To conduct cost-of-illness, cost-effectiveness and budget impact analyses of the task-sharing approach to echocardiographic detection for early detection and management of RHD in Australia and Timor-Leste.

## Methods

### Study design

NEARER SCAN is a type II effectiveness-implementation hybrid study, evaluating implementation of an intervention designed to achieve early detection and management of RHD in high-risk populations. The study will run from August 2022 to June 2025. A package of implementation strategies will be co-designed at each site with the local community and participating primary healthcare service around non-adaptable elements of the intervention (table 1), and mapped onto an initial Theory of Change framework. Co-design, implementation and evaluation will occur simultaneously, and we will evaluate both the implementation strategy and the clinical effectiveness of the intervention.^36^

**Table 1:** The non-adaptable functions of the active case finding programme and some of the adaptable forms of the active case finding programme to be locally tailored during the co-design process

Function (non-adaptable)	Form (adaptable)
Creating a community-led implementation group that provides feedback throughout the study	Size, membership and style of consultation with community-led implementation group (eg, meeting together or individual discussions)
Training local staff to acquire echocardiogram images	Who does the active case finding (eg, professional backgrounds of the staff trained, number of staff members trained)
Using Philips Lumify hand-held device	When the active case finding occurs (eg, annual check-ups, set days per month, community events)
Using defined SPLASH protocol	Where the active case finding occurs (eg, at the clinic, in school, house visits, outstations)
Cardiologists reviewing images	Defining what constitutes a successful programme at each site
Entering data into REDCap and cardiologists entering structured report into Tricefy	The implementation strategies to assist integration at each site
Linking positive screens into the cascade of care	

SPLASHSingle Parasternal Long Axis view with a Sweep of the Heart

### Setting and population

The study will be carried out in approximately 10 primary healthcare and antenatal care settings across Timor-Leste and Australia’s Northern Territory and Western Australia. Stakeholder engagement and discussions underpinning the approaches to be used in this study have occurred over several years at participating sites in Australia and Timor-Leste. Community members have identified RHD as a significant priority and have sought out initiatives targeting early case detection and management. Extensive consultations have resulted in requests to transition echocardiographic detection from isolated research activities into implementation of a co-designed RHD active case finding programme within communities.

The target population in each community includes children and young people aged 5–20 years and pregnant women, representing high-risk subpopulations for RHD. In these culturally and linguistically diverse settings, native language speakers, interpreters and training and community health promotion materials in local languages will be used, relevant to each site.

### Governance

Strategic input for the study will be provided by a newly established Indigenous Advisory Group (specific for the NEARER SCAN project) and the existing Menzies Timor-Leste Strategic Reference Group. Communities will also be asked to nominate individuals for a community-led implementation group who will provide ongoing local governance and feedback to the community.

### Co-design

Co-design in this study builds on more than 5 years of informal conversations between communities, clinics and study authors, developing the programme idea to directly meet the requests from communities to have local healthcare workers trained to detect RHD in their own community. Co-design is a collaborative process whereby local stakeholders contribute to each phase of the study. Co-design has the potential to create a shared understanding of the intervention, identify and meet needs, shift power dynamics to enable community ownership and develop trust and confidence in the research process.^37^ The co-design process is intended to achieve a more successful implementation of the active case finding programme and to see that the research findings are used in an effective and sustainable way. The process will engage the participating primary healthcare service as well as community stakeholders including traditional owners, community leaders, people with RHD and their families, school representatives, and representatives from other relevant community groups.^38^ Discussions will take various forms (informal and formal, yarning,^39^ individual and group discussions) and occur over several visits. The process will explore community-specific aspects of the implementation, existing health service delivery workflow, selection of staff to be trained and the associated human and technology resource capacities, governance structures and readiness of service for change.

**Figure 1:**
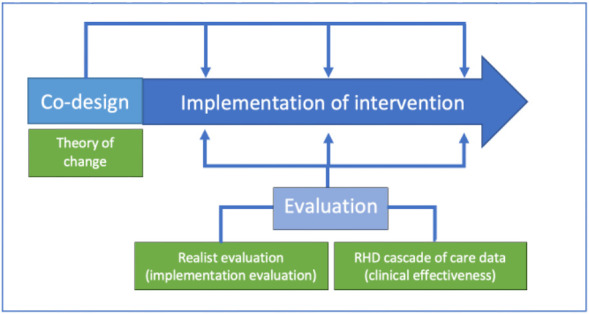
The Non-Expert Acquisition and Remote Expert Review of Screening echocardiography images from Child health and AnteNatal clinics (NEARER SCAN) study design. Blue: study components. Green: methodologies. RHD, rheumatic heart disease.

A Theory of Change approach will be used in the initial co-design process to build consensus and capture the tailored adaptions and the logic for how and why these adaptions are expected to work in each site.^40^,^43^ The approach will focus on hidden assumptions and anticipated causal mechanisms to identify insights that may be useful for planning scale-up and wider adoption of a similar programme in other settings.^44^,^48^ This work will inform the subsequent realist evaluation which will consider the adaptions made over time as the programme is implemented based on feedback from each local community-led implementation group (figure 1).

As some aspects of the programme may look different in each site in accordance with the co-design process, careful consideration has been taken to ensure evaluation integrity across the sites while allowing for flexibility in the delivery of the intervention. Our intention in implementing this complex intervention is that integrity should be assessed on the fidelity to the intervention’s *function* (the fundamental idea underpinning the programme and the theory of how that idea will lead to the programme’s aims being achieved) and to the standardised clinical active case finding programme pathway, rather than to the precise *form* of the intervention (tailored for each site in the co-design process) that will be used to achieve these functions.^49^ Table 1 highlights the function and form of NEARER SCAN which provides guidance for each site on what can be adjusted for context without undermining the core programme idea (and thus maintaining the integrity of the evaluation).

### Implementation activities

#### SPLASH echocardiography training for non-expert acquisition

SPLASH echocardiogram is a highly abbreviated imaging protocol used to screen for RHD in high-risk populations.^50^ It has been demonstrated to be accurate for the detection of RHD and can be taught to non-experts in the context of echocardiographic RHD screening.^35^ It can be performed in routine clinical care settings, allows for modesty, does not require removal of clothing and takes approximately 5–10 min to perform. Using a training course and resources that have been developed and tailored in collaboration with communities,^28^ non-expert practitioners from the participating communities will be trained to perform SPLASH echocardiography. The training course includes online training modules, in-person lectures, workshops and practical training (across approximately three full days or five half days), expert supervised SPLASH echocardiograms (target number of SPLASH echocardiograms: 100) and theoretical and practical skill assessments (at the completion of the supervised 100 scans). Each community-led implementation group will partner with the study team to ensure culturally appropriate local customisation of the training course, for example, language, metaphors, prioritisation of training local community members, linkage to other training or health promotion activities.

Healthcare staff from each site (approximately 4–8 per site depending on interest and feasibility) will undertake training in SPLASH echocardiography, with the aim of participating in subsequent active case finding activities. Local healthcare workers are anticipated to include a mix of Aboriginal health practitioners and workers, general practitioners, remote area nurses, midwives and general sonographers. The training will be delivered by trainers with expertise in echocardiographic detection for RHD, including cardiologists and cardiac sonographers.

#### Embedding task-sharing of SPLASH echocardiography into healthcare delivery

**Figure 2:**
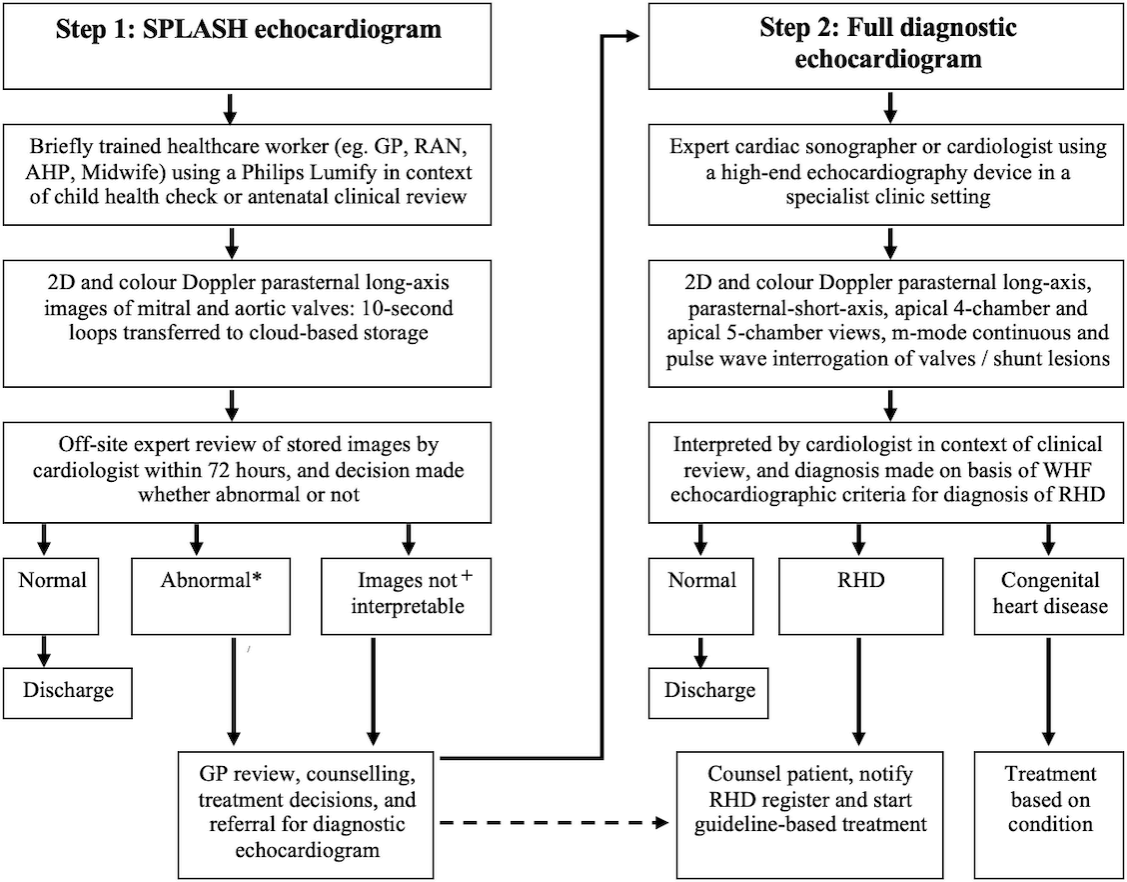
Non-Expert Acquisition and Remote Expert Review of Screening echocardiography images from Child health and AnteNatal clinics (NEARER SCAN) active case finding programme flow chart. *Abnormal SPLASH echocardiogram defined below. Diagnosis of RHD will be based on the revised World Heart Federation (WHF) minimum echocardiographic criteria for RHD. +If initial SPLASH is uninterpretable, educational and training feedback with be given to the non-expert by the expert reviewer. A repeat SPLASH will then be performed. If repeat SPLASH images are abnormal, or again not interpretable, the participant will be referred for a diagnostic echocardiogram. AHP, Aboriginal health practitioner; GP, general practitioner; RAN, remote area nurse; RHD, rheumatic heart disease; SPLASH, Single Parasternal Long Axis view with a Sweep of the Heart.

The co-design process will be used to determine exact details as to when and where SPLASH echocardiography will be conducted at each site. The anticipated process for SPLASH echocardiography task-sharing is shown in figure 2.

A SPLASH echocardiogram will be performed by a trained local healthcare worker using a Philips Lumify hand-held echocardiographic device. The SPLASH images will be uploaded to Tricefy, which is a cloud-based medical image communication application (California, USA). Images will be accompanied by identifiable participant data (name, date of birth, medical record number) and the local healthcare worker’s name. Healthcare workers will have the option to add a comment that includes their interpretation of the image findings, or other relevant clinical information. The uploaded images will then be reviewed and reported by an off-site cardiologist with expertise in RHD (referred to as an expert for study purposes) within 72 hours. The cardiologist will decide whether the images are normal, abnormal or uninterpretable according to the definitions in online supplemental material (Definitions of abnormal Single Parasternal Long Axis view with a Sweep of the Heart (SPLASH) echocardiogram),^21^ uploading their interpretation, recommendations and any additional notes in a report that will be accessible to healthcare staff on Tricefy.

Additional mechanisms for push notification of SPLASH results will be developed with individual sites. These may include integration with existing electronic medical record systems, automated emails or use of secure phone messaging services such as WhatsApp or Signal. In addition, healthcare workers will be able to contact an on-call remote expert for discussion of SPLASH findings and advice regarding next steps. Non-experts will receive ongoing supervision and feedback from reviewing experts on image acquisition quality and image findings, and uninterpretable images will be reattempted. Participants with abnormal or repeated uninterpretable images should be referred by the local healthcare service for a full diagnostic echocardiogram.

Treatment decisions will usually be made following a full diagnostic echocardiogram and specialist review. If, however, the SPLASH findings are strongly suggestive of RHD, or there are other clinical indications for treatment, the primary healthcare team may choose to initiate counselling, treatment (including for suspected RHD) and/or additional referrals before completion of the full diagnostic echocardiogram.^51^

Any new case of RHD will be notified to the relevant local or jurisdictional RHD register, based on national guidelines in Australia and Timor-Leste.

Data audits will be conducted at least monthly, and if abnormal or uninterpretable SPLASH images have not been appropriately actioned, a reminder will be sent to the relevant healthcare service as a safety net in the context of this study. Referrals or treatment that occur as a result of these reminders will be evaluated separately to those that are generated as a result of the primary healthcare service response to the initial report.

### Participation and recruitment

Eligible participants are children and young people aged 5–20 years residing in participating communities, and pregnant women at high risk for RHD. Pregnant women are considered to be at high risk if they are Aboriginal or Torres Strait Islander, or if they live in a remote community in northern Australia, or if they have immigrated to Australia from a high-prevalence country with all-age RHD prevalence >1 per 1000 people, or if they live in Timor-Leste. Women, children and young people with known history of ARF, RHD or other existing cardiac disease will not be excluded.

Pathways for recruiting eligible participants will be co-designed with each local community and participating primary healthcare service. We anticipate that this will primarily involve scanning opportunistically in the primary healthcare setting, with occasional organised screening days at local schools or community centres. In some settings, scanning may occasionally occur in the neighbourhoods as guided by trained local staff.

### Patient and public involvement

The conceptualisation of this study was shaped by patients’ priorities, with a focus on improving early detection of RHD and addressing culturally relevant care. Patients with lived experience and their families will participate in the implementation co-design workshops to ensure the implementation and evaluation align with their needs and preferences. Results of the final study will be disseminated through community meetings and accessible written and visual summaries provided to participating clinics and community groups.

### Evaluation

#### Evaluation of implementation

The evaluation of the implementation of the programme will be a mixed-methods theory-based evaluation. A theory-based evaluation has been chosen for this evaluation as it allows for an understanding of *if* the implementation works in real-world everyday practice, and assists to explain differences in its success across sites. This will include a realist evaluation, which is an applied, explanatory approach to evaluation that seeks to answer practical questions of what works for whom, under what circumstances and why.^52^ Realist evaluations are increasingly being used in studies of complex interventions and are recommended in the Australian government’s 2020 *‘Guide to Evaluation under the Indigenous Evaluation Strategy’*^53^ and the UK Medical Research Council’s 2021 *‘Framework for Developing and Evaluating Complex Interventions’*.^54^ The realist evaluation approach acknowledges that the intervention is influenced by context and seeks to identify and explain variations across settings. Outcomes of the implementation evaluation are shown in table 2.

**Table 2:** Implementation and economic evaluation outcome measures

Outcome measure	Data collection	Data analysis
Adoption	Provider interviews Administrator interviews Observation	Primary qualitative data will be analysed thematically using NVivo software.Primary quantitative data will be analysed with descriptive statistics.Secondary analysis of other data collected in NEARER SCAN study may also be used to develop, test and refine realist programme theories.
Penetration	Provider interviews Administrator interviews Observation Usage data on REDCap	
Sustainability	Normalisation Measure Development Questionnaire (NoMAD) Usage data on REDCap Administrator interviews Provider interviews Observation	
Fidelity	Theory of Change and follow-up checklist of indicators and observation	
Health systems costs and economic evaluation of implementation	Study budget Cost-of-illness surveys	Budget impact analysisCost-effectiveness analysis

NEARER SCANNon-Expert Acquisition and Remote Expert Review of Screening echocardiography images from Child health and AnteNatal clinics

#### Data collection for the implementation evaluation

Qualitative and quantitative data will be collected for the implementation evaluation. The development of testable realist initial programme theories will occur using data from the co-design, Theory of Change work, a document review, narrative review and informal discussions with international RHD active case finding experts. For the realist evaluation (table 2), the co-design process will inform what data will be collected and how, as well as outcome measures indicating successful implementation. Community-directed metrics of programme success may result in an expanded set of outcome measures. It is anticipated that data collection methods may include interviews, surveys, focus groups and observational field notes. All data collected in the NEARER SCAN study will also contribute to testing and refining the programme theories of the realist evaluation.

#### Qualitative data analysis

Qualitative data analysis will be conducted using a framework approach for the Theory of Change and a process of retroduction using abductive reasoning for the realist evaluation (ie, identifying the most likely explanation for each set of observations). Data will be organised using QSR NVivo V.12,^55^ ^55^ stored on a secure server at Menzies School of Health Research (Darwin, Australia). These data will be mapped to different nodes within NVivo which will be grouped for each community involved in the study. The coding will be set up in a way to allow for identification of themes across communities.

#### Evaluation of clinical effectiveness

Clinical effectiveness of the intervention will be evaluated based on analysis of impact on key clinical outcome measures. The primary outcome for evaluation of clinical effectiveness is change in proportion of the target population prescribed secondary prophylaxis for RHD by the end of the study compared with a baseline timepoint prior to the date of echocardiographic active case finding (table 3).

**Table 3:** Clinical effectiveness evaluation outcome measures

Outcome measure	Data collection	Data variables	Data analysis
**Primary outcome**			
Change in the proportion of the target population who are prescribed secondary prophylaxis based on national (Australia or Timor-Leste) RHD guidelines	Primary healthcare records Local RHD register	Initial BPG prescriptionInitial BPG delivery	Descriptive statisticsTwo-tailed comparison of proportions
**Secondary outcomes**			
Proportion of people with an abnormal or uninterpretable screening echocardiogram who get a full diagnostic echocardiogram within 3 months and receive a diagnosis	Primary healthcare records Local cardiology/echocardiography service provider records and primary healthcare records	Echocardiography referralEchocardiography completedCardiology review completedHealthcare worker interaction for diagnosis	Descriptive statistics
Proportion of people diagnosed with RHD who are retained in the cascade of care	Primary healthcare records Cardiology/specialist service provider records	GP interaction re RHD recordedSpecialist referral generatedSpecialist review completed	Descriptive statistics
Proportion of people prescribed secondary prophylaxis who receive ≥80% indicated doses in the 12 months prior to the end date of the study	Primary healthcare records Local RHD register	BPG prescriptionBPG delivery	Descriptive statistics
Description of antenatal and birthing care, and maternal and birth outcomes in women diagnosed with RHD on echocardiographic screening	Hospital and primary healthcare records	Location of deliveryMode of deliveryICU admissionNICU admissionPeripartum cardiovascular complication	Descriptive statistics
Prevalence of RHD and distribution of severity in children and pregnant women	Study database Primary healthcare records Local RHD register	Disease severity RHD diagnosis	Descriptive statistics

.BPG, benzathine penicillin G; GPgeneral practitionerICU, intensive care unit; NICU, neonatal intensive care unit; RHD, rheumatic heart disease

The primary outcome will be reported for all communities as well as aggregated results for Australia and Timor-Leste. This primary outcome represents a clinically meaningful outcome demonstrating whether active case finding for RHD results in a change in practice at the individual patient level that has a measurable public health impact in terms of increased access to guideline-based care. The proportion of the population prescribed secondary prophylaxis is expected to increase with detection of new RHD cases but may also be reduced as people complete treatment. The number of people in each community who are commenced on secondary prophylaxis and the number of people in each community who cease secondary prophylaxis during the study period will be collected as secondary outcomes. Other secondary outcomes include elements of the cascade of care from diagnosis to antibiotic adherence (online supplemental material (Cascade of Care).

This study will also report on maternal and birth outcomes in women found to have RHD on echocardiographic screening, and prevalence of RHD in screened populations (table 3).

#### Sample size calculation

The sample size has been calculated based on the primary outcome measure. To observe an absolute increase of 2.4% of the target population prescribed secondary prophylaxis, from a baseline prevalence of 4.4% to 6.8% (based on previous data from the Northern Territory^3^), with 95% two-sided significance and 80% power, the sample size required is 1421. However, if a lower proportion of the target population is prescribed secondary prophylaxis at baseline (as expected in Western Australia and Timor-Leste), then the power of the study is increased. The study will aim to enrol >1500 participants (children and pregnant women) across approximately 10 sites (~150 participants per site); five sites in Australia and five sites in Timor-Leste over 18 months.

#### Clinical effectiveness data collection

Study data will be collected using a REDCap V.13 (Vanderbilt University, USA) database,^56 57^ hosted at Menzies School of Health Research (Darwin, Australia). Data relating to the expert reviewer interpretation of SPLASH images will be entered into REDCap. Expert reviewers will also enter a brief, structured report into Tricefy that can be seen by the healthcare workers and used as part of the continued education and supervision process. The formal diagnostic echocardiogram will be performed by local service providers and images will be stored on their equipment or server based on their own policies and procedures.

Demographic and clinical data will be obtained from primary healthcare and RHD register data systems by research staff, who will enter data into REDCap. Secondary data will include: participant demographics, obstetric history (for pregnant women), history of known ARF/RHD, clinical management decisions including referrals for formal echocardiography and commencement of secondary prophylaxis, time taken for SPLASH echocardiography to be performed, formal echocardiography findings and progression through the RHD cascade of care.

#### Quantitative data analysis

Quantitative analysis of clinical effectiveness data will be conducted using STATA V.18 (StataCorp, USA). The proportion of the target population prescribed secondary prophylaxis will be calculated prior to recruitment and screening participants, and at completion of the study, and will be described with 95% CIs. Numerator data will be obtained from clinic and RHD register data; the denominator will be based on census population data or health information system population data, following consultation with the community-led implementation group regarding the most accurate source for local population estimates.

The absolute change in proportion of the target population prescribed prophylaxis will be calculated with adjustment for clustering by community and described with 95% CIs. Proportions from commencement and completion of the study will be compared using a two-tailed comparison of proportions test. A p value <0.05 will be considered significant.

Descriptive analyses will be used to quantify secondary outcomes on the epidemiology of RHD in the target population at completion of the study. The proportion of enrolled participants progressing through each stage of the RHD cascade of care will be calculated (with 95% CI). Prevalence of RHD (and 95% CI) will be calculated for the different participating communities and jurisdictions, stratified by sex and age group, based on proportion of people screened who have a diagnosis of RHD. Prevalence of known RHD will also be estimated by calculating the proportion of people in the community with a diagnosis of RHD entered on the relevant register at completion of the study.

#### Economic evaluation

The economic evaluation component aims to provide the data and analyses required to aid decision makers in assessing future funding required for active case finding, such as support through the Australian Medicare Benefits Scheme or other sources of government funding. The evaluation will comprise a cost-of-illness study and model-based cost-effectiveness and cost-benefit analyses.

The cost-of-illness study will focus on the costs of implementing active case finding, including training, scanning and expert review. It will also capture the costs of diagnosing and managing screen-positive cases in the short term (ie, within the study period), including the cost of referral for full cardiology examination at a regional health centre (eg, Royal Darwin Hospital) or by outreach, as previous research has indicated that this cost is an important driver of active case finding cost-effectiveness.^58^ The costs associated with active case finding and with RHD diagnosis and short-term management will be summarised using descriptive statistics and aggregated to provide an overall estimate of the cost of active case finding and the economic burden of RHD.

The cost-effectiveness analysis will evaluate the value for money in diagnosing and managing RHD afforded to the trialled active case finding protocols compared with alternative methods, such as diagnosis by auscultation during a child health check or by onset of clinical disease. A model-based approach will be used, drawing on existing models,^58 59^ to extrapolate the outcomes beyond the study time frame. Outcomes will include predicted changes in RHD progression using previous longitudinal analyses for baseline progression,^9^ changes in disability-adjusted life-years (DALYs) lost to ARF and RHD and incremental cost-effectiveness ratios (ie, cost per DALY saved). Sensitivity analysis will be conducted to assess the uncertainty in model assumptions and parameter estimates.

The budget impact analysis will estimate the financial considerations of implementing active case finding by the trialled protocols from different public funding providers, such as Medicare Benefits Scheme reimbursement for individual screens. A model will be developed to simulate the expected changes in net costs to each funding provider. Factors such as active case finding coverage, diagnostic referral rates, and short- and long-term treatment costs, potentially offset against the cost of severe disease, will be considered.

## Discussion

The NEARER SCAN study addresses the major clinical issue of RHD underdiagnosis using pragmatic, novel and culturally appropriate strategies. This study directly responds to the requests of participating communities to co-design a task-sharing active case finding programme for RHD and implement this within routine service delivery. The study design moves away from traditional large-scale, short-term RHD active case finding programmes and introduces a potentially sustainable active case finding strategy using task-sharing, telemedicine and a hand-held device integrated into routine healthcare service. This will be evaluated as a complex intervention, rather than tested with a randomised trial design.

The study will evaluate both the clinical effectiveness and implementation of an intervention addressing the need for early detection and management of RHD in endemic settings. The study will also provide information regarding the prevalence and disease severity in these populations that can be used to support health service funding and development. Evaluation has been designed to ensure practical, useful learnings so that the active case finding programme can be adapted, implemented and scaled in other endemic regions.

Other potential improved outcomes from the intervention include increased community engagement and proactivity around retention in RHD guideline-based care. This, in turn, may demonstrate the benefits of engaging local community in research programmes impacting individual community groups.

### Limitations and strengths

The choice of study design was influenced by the complex health systems involved. It is appropriate to implement strategies that are acceptable and sustainable for health structures that serve diverse populations. While local tailoring may limit the ability to directly replicate the programme in other settings, the study may also identify aspects of the co-design process that could be important to consider in different settings.

The primary clinical effectiveness outcome has been chosen to measure the impact of the intervention on clinically meaningful changes in practice. However, changes in the primary outcome are likely to reflect the overall study implementation and may also be influenced by factors other than the intervention. It will not be possible to completely separate out the impact of SPLASH echocardiography on its own. However, prior research supports that the approach to community-led development is likely to be important and that any echocardiographic active case finding programme should be scaffolded by such resources.^60^

Some members of the implementation team will also be involved in the evaluation of the study, which may introduce some bias into the evaluation. However, these dual roles are anticipated to enable the programme to adapt swiftly to accommodate lessons learnt, which is a significant priority underpinning the co-design approach to this implementation study. Experts who are involved in the implementation evaluation will not contribute to off-site review of SPLASH echocardiographic images.

### Data sovereignty and dissemination of findings

The NEARER SCAN study has been established based on a commitment to principles of Indigenous Data Sovereignty,^61^ and the investigators will be accountable to the Indigenous Advisory Group and the Menzies Timor-Leste Strategic Reference Group, as well as to individual community-led implementation groups in each site. These groups will be involved in the local dissemination of the findings, which will occur through verbal and written summaries. Communities will be encouraged to use these data for the well-being of their people. A summary of results will be presented in written form (English and Tetum) to the Ministry of Health in Timor-Leste, and to the Timor-Leste National Institute of Public Health, as well as to members of the Northern Territory Department of Health and the Western Australia Department of Health in Australia. Findings will also be presented at academic meetings and published in peer-reviewed journals.

## Conclusion

The NEARER SCAN study will work with communities in Australia and Timor-Leste to co-design and implement a model of echocardiographic active case finding that includes training for local healthcare workers and support for task-sharing. This evaluation will assess the clinical effectiveness of the intervention as well as the implementation. This will be used to create an evidence-based template for scaling up this intervention in other similar settings.

## Ethics approval and consent to participate

This study involving human participants complies with the Declaration of Helsinki and in accordance with relevant guidelines and regulations. Ethical approval has been obtained from the Human Research Ethics Committee of the NT Department of Health and Menzies School of Health Research, Darwin (HREC-2022-4479), the Western Australian Aboriginal Health Ethics Committee (HREC-1237) and the Instituto Nasional Saude Publika Timor-Leste Ethics and Technical Committee (03-UEPD/INSP-TL/V/2023). Study results will be disseminated in the communities involved in the study and through peer-reviewed publications and conference abstracts.

Information regarding the active case finding programme and the evaluation will be provided in local languages at each site. A script that outlines key information required for potential participants to provide informed consent for both the SPLASH and having their deidentified data collected as part of the research will be used. All participants require informed consent to be enrolled. Ethics approval for verbal informed consent has been granted in Timor-Leste (Instituto Nasional Saude Publika Timor-Leste Ethics and Technical Committee (03-UEPD/INSP-TL/V/2023)) and the Northern Territory (Human Research Ethics Committee of the NT Department of Health and Menzies School of Health Research, Darwin (HREC-2022-4479)); written informed consent will be required in Western Australia. Informed consent for participation in the active case finding programme will be obtained from a parent or guardian for those aged less than 16 years.

Informed consent will also be obtained prior to participation in interviews and discussions, which will be used to inform qualitative evaluation of implementation.

## supplementary material

10.1136/bmjopen-2023-083467 online supplemental file 1

10.1136/bmjopen-2023-083467 online supplemental file 2
