# Supplementary material for: NEARER SCAN (LENO BESIK) evaluation of a task-sharing echocardiographic active case finding programme for rheumatic heart disease in Australia and Timor-Leste: protocol for a hybrid type II effectiveness-implementation study
Source: BMJ Open. 2024 Oct 18;14(10):e083467. doi: 10.1136/bmjopen-2023-083467 (PMC11492941; doi:10.1136/bmjopen-2023-083467)
Supplement: online supplemental file 1 [file bmjopen-14-10-s001.pdf]

Supplemental Material. Definitions of abnormal Single Parasternal Long Axis view with a Sweep of the Heart (SPLASH) echocardiogram (21)

**SPLASH to be reported as abnormal if any of the following are present:**	
Mitral regurgitation (must meet both criteria)	1. Mitral regurgitation jet length ≥ 15mm (< 30 kg) or ≥ 20mm (≥ 30 kg)<br>2. Seen in ≥ 2 consecutive frames
Aortic regurgitation (must meet both criteria)	1. Any aortic regurgitation jet length<br>2. Seen in ≥ 2 consecutive frames
Mitral stenosis	Restricted leaflet motion with reduced valve opening
Other abnormalities that will be reported and referred for formal echocardiogram if identified	1. Any intra-cardiac shunt or other congenital abnormality, OR<br>2. Impaired ventricular systolic function based on visual assessment, OR<br>3. Dilated ventricle based on visual assessment, OR<br>4. Visual evidence of elevated right ventricular volume or pressure loading, OR<br>5. Tricuspid regurgitation jet length ≥ 20mm and seen in ≥ 2 frames
