# Supplementary figures and images for: NEARER SCAN (LENO BESIK) evaluation of a task-sharing echocardiographic active case finding programme for rheumatic heart disease in Australia and Timor-Leste: protocol for a hybrid type II effectiveness-implementation study
Source: BMJ Open. 2024 Oct 18;14(10):e083467. doi: 10.1136/bmjopen-2023-083467 (PMC11492941; doi:10.1136/bmjopen-2023-083467)

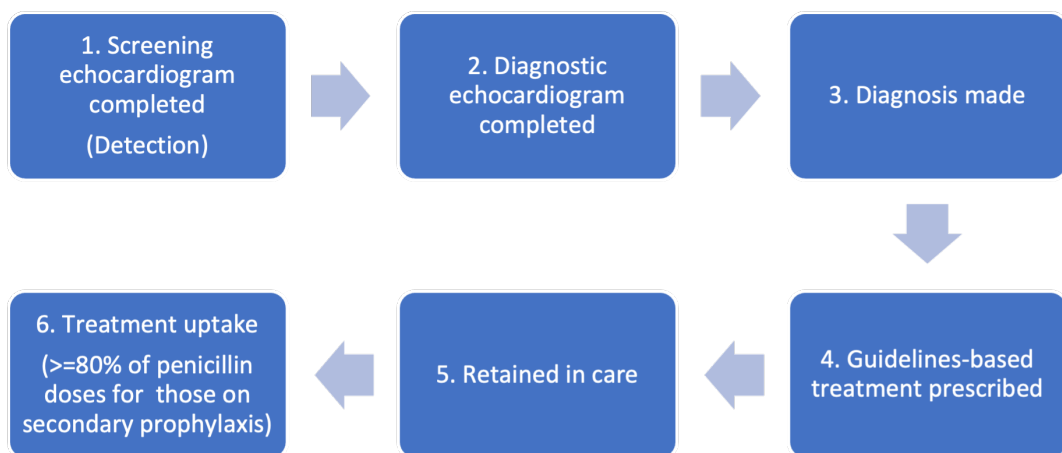

Supplement: online supplemental file 2 [file bmjopen-14-10-s002.pdf]
